# Sex-Specific Association between Serum 25-Hydroxyvitamin D and Metabolic Risk Factors in T2DM Patients

**DOI:** 10.1155/2020/9238719

**Published:** 2020-06-04

**Authors:** Xiaomin Sun, Sirui Zhou, Xin He, Youfa Wang, Wei Cui

**Affiliations:** ^1^Global Health Institute, School of Public Health, Xi'an Jiaotong University Health Science Center, 76 West Yanta Road, Xi'an, Shaanxi 710061, China; ^2^Department of Geriatric Endocrinology, First Affiliated Hospital, Xi'an Jiaotong University Health Science Center, 76 West Yanta Road, Xi'an, Shaanxi 710061, China; ^3^Department of Endocrinology, Xi'an Aerospace General Hospital, 159 Jitai Road, Yanta District, Xi'an, Shaanxi 710061, China

## Abstract

**Objective:**

To evaluate the relationship between serum 25-hydroxyvitamin (25(OH)D) levels and metabolic risk factor levels in patients with type 2 diabetes mellitus (T2DM) on a sex-specific basis.

**Methods:**

Our study comprised 507 patients with T2DM (321 men, 186 women; median age, 59 years). The metabolic risk factors examined included lipoprotein(a), glycated albumin (GA-L), and random blood glucose (RBG); the levels of these parameters were determined enzymatically. Serum 25(OH)D levels were measured by commercial ELISA kits. Participants were divided into low and high 25(OH)D groups according to the median 25(OH)D concentration (13.2 ng/mL). Two-way analysis of covariance and multiple linear regression analysis were performed.

**Results:**

The median 25(OH)D concentration was 13.9 ng/mL in men and 12.2 ng/mL in women. 25(OH)D levels inversely correlated with levels of three metabolic risk factors in a sex-dependent manner after adjusting for several confounding factors. These were lipoprotein(a) in men (141.3 ± 137.9 and 195.3 ± 204.8 mg/L in high and low 25(OH)D groups, respectively; *P* < 0.05); GA-L in women (22.2 ± 8.8 vs. 23.3 ± 7.3% in high and low 25(OH)D groups, respectively; *P* < 0.05). In a subgroup analysis, serum 25(OH)D levels inversely correlated with lipoprotein(a) levels in men (*β* = −0.185, *P* = 0.002) and RBG levels in women (*β* = −0.176, *P* = 0.028).

**Conclusion:**

Higher serum 25(OH)D levels indicate a more favorable lipid profile in men and a more favorable glucose profile in women.

## 1. Introduction

Type 2 diabetes mellitus (T2DM) is a global public health concern that threatens the economies of all nations and developing countries in particular [1]. In a nationally representative survey in mainland China, the estimated overall prevalence of diabetes was 10.9%, with 60% of diabetic patients unaware of their diagnosis [2]. In addition, more than 50% of Chinese people with glucose metabolism disorders are vitamin D-deficient [3].

Vitamin D regulates calcium and phosphorus homeostasis, and in the last few decades, its receptors have been observed in several types of insulin-sensitive tissue, such as muscle, fat, and hepatic tissue [4]. Mounting evidence implicates a low level of 25-hydroxyvitamin D (25(OH)D), the storage form of vitamin D and the form used in the evaluation of vitamin D status, has been associated with high incidence of T2DM and metabolic syndrome [5, 6].

In the Third National Health and Nutrition Examination Survey, the levels of 25(OH)D negatively correlated with insulin resistance in non-Hispanic whites and Mexican Americans but not African-Americans [7]. These findings suggest that this relationship is ethnicity-dependent. However, there are limited studies evaluating the relationship between 25(OH)D concentrations and metabolic risk factors in Chinese populations [8–14], especially in T2DM patients [12–14]. Lu et al. [8] linked this relationship to glucose control in their study of individuals in East China. However, sex-specific differences have not been explored, although several studies indicate that the association between 25(OH)D levels and metabolic risk was stronger in men than women [9, 10]. The objective of our study was to evaluate the relationship between serum 25(OH)D and metabolic risk factors, and whether the relationships differ by sex in T2DM patients in China.

## 2. Materials and Methods

### 2.1. Subjects

The study recruited T2DM patients from the First Affiliated Hospital of Xi'an Jiaotong University. Subjects were excluded if they had missing laboratory (*n* = 40) or personal (*n* = 38) data, were younger than 18 years old, (*n* = 2), or were taking calcium or vitamin D supplements (*n* = 9). Finally, a total of 507 subjects were enrolled. Among these, 251 (49.5%) had received insulin treatment, 236 (46.5%) had taken antihypertensive drugs, and 81 (16.0%) had taken lipid-lowering drugs. Analyses involving glycated albumin (GA-L) levels were conducted on 449 subjects only (285 men and 164 women) owing to lack of data for the remaining subjects. The study was approved by the ethics committee at Xi'an Jiaotong University Health Science Center. Written consent was obtained from all participants, and the procedures were implemented in accordance with the approved guidelines.

### 2.2. Measurements

T2DM was defined as self-reported diabetes previously diagnosed by health-care professionals and was confirmed via a validated supplementary questionnaire that assessed blood test, symptoms, and therapy. Patients whose glycosylated hemoglobin (HbA1c) levels were ≥6.5% in a serological test in this study, but were <6.5% previously, were considered to have newly diagnosed T2DM as defined by the American Diabetes Association [11].

The following demographic information was collected from the medical records by the medical staff: age, sex, height, weight, treatment time, marital status, smoking status, alcohol use, insulin use, family history of diabetes, and diabetes duration. Body mass index (BMI) was calculated by dividing the body mass in kilograms by the square of the height in meters (kg/m^2^). Systolic blood pressure and diastolic blood pressure were measured by accredited nurses.

The metabolic risk factors examined in our study were random blood glucose (RBG), GA-L, HbA1c, lipoprotein(a), total cholesterol (Total-C), low-density lipoprotein cholesterol (LDL-C), high-density lipoprotein cholesterol (HDL-C), triglycerides, and apolipoprotein (Apo) A, ApoB, and ApoE. Blood samples for measurements of all factors except RBG and GA-L were obtained from the patients after they had fasted for at least 8 hours. HbA1c levels were measured via high-performance liquid chromatography (Bio-Rad Variant II; Bio-Rad, Hercules, CA, USA). Levels of the remaining risk factors were determined enzymatically using an automated analyzer (LABOSPECT 008; Hitachi High-Technologies, Tokyo, Japan). ApoA/ApoB and HDL-C/LDL-C ratios were calculated. Serum 25(OH)D concentrations were determined using an electrochemiluminescence assay (Roche Diagnostics GmbH, Mannheim, Germany). High and low 25(OH)D group were divided according to the median values of 25(OH)D levels (13.2 ng/mL), respectively. The intra- and interassay coefficients of variability were both <10% for 25(OH)D levels.

### 2.3. Statistical Analysis

All statistical analyses were performed using SPSS software, version 22.0 (SPSS, Inc., Chicago, IL, USA). The Kolmogorov–Smirnov test was used to assess the normality of data distribution; the values for 25(OH)D, RBG, HbA1c, ApoB, ApoE, lipoprotein(a), Total-C, LDL-C, HDL-C, and triglycerides were log-transformed prior to analysis. Student's *t*-test (for normally distributed variables) and the Mann–Whitney *U*-test (for nonnormally distributed variables) were used to evaluate the significance of differences between the sex. The influence of sex and 25(OH)D concentration on the levels of the metabolic risk factors was assessed via two-way analysis of covariance (ANCOVA) adjusted for the appropriate covariates. A post hoc test with the Bonferroni correction was used to identify significant differences if a significant main effect or interaction was observed. Normally distributed variables are presented as mean ± standard deviation, skewed variables as median (interquartile range), and categorical variables as percentages, unless otherwise indicated. The statistical significance level was set at *P* < 0.05.

## 3. Results

The median age of the 507 T2DM patients in our study was 59 years, and 321 (63.3%) patients were men. The percentage of patients with vitamin D deficiency (<20 ng/mL) and vitamin D insufficiency (20–30 ng/mL) was 78.5% and 18.9%, respectively (Supplemental Table 1). The median 25(OH)D level was 13.9 ng/mL in men and 12.2 ng/L in women.

Women weighed less than did men and were shorter (both *P* values < 0.05) (Table 1). They also had lower BMIs and diastolic blood pressures and higher ApoA, ApoE, Total-C, HDL-C, and phosphorus levels and ApoA/ApoB ratios (all *P* values < 0.05). There were no other significant difference between men and women.

**Figure 1:**
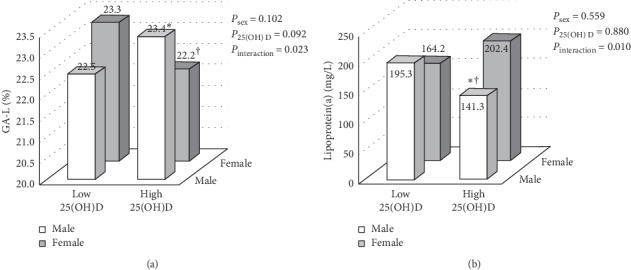
Joint association of 25(OH)D levels and sex on lipoprotein(a) and glycated albumin (GA-L). The values for lipoprotein (a) and GA-L were log-transformed for two-way analysis of covariance. The analysis was adjusted for age, body mass index, season, marital status, smoking status, alcohol use, insulin use, family history of diabetes, and diabetes duration. ^#^GA-L, *n* = 449. ^*∗*^*P* < 0.05 vs. women in the same 25(OH)D group. ^†^*P* < 0.05 vs. low 25(OH)D in the same sex group.

To identify interactions between 25(OH)D and sex on metabolic risk factors, two-way ANCOVA was performed after adjusting for age, BMI, season, marital status, smoking status, alcohol use, insulin use, family history of diabetes, and diabetes duration (Table 2 and Figure 1). Significant interactions between 25(OH)D and sex on lipoprotein(a) and GA-L were observed (*P* < 0.05). High 25(OH)D levels correlated with favorable lipoprotein(a) levels in men (141.3 ± 137.9 and 195.3 ± 204.8 mg/L for high and low 25(OH)D groups, respectively; *P* < 0.05). High 25(OH) D levels correlated with low GA-L levels in women (2.2 ± 8.8% and 23.3 ± 7.3% for high and low 25(OH)D groups, respectively; *P* < 0.05).

Because the association between 25(OH)D and metabolic risk factors differed according to sex, we performed separate multiple linear regression analyses in men and women (Table 3). As shown in Model 1, 25(OH)D levels negatively correlated with lipoprotein(a) levels in men (*β* = −0.203, *P* < 0.001) and RBG levels in women (*β* = −0.150, *P*=0.041). These relationships remained significant after adjusting for several confounding factors including BMI (Models 2 and 3).

## 4. Discussion

The present study shows sex-specific correlations between 25(OH)D levels and the levels of three metabolic risk factors (lipoprotein(a), GA-L, and RBG) in T2DM patients. High serum 25(OH)D levels indicated a more favorable lipid profile in men and a more favorable glucose profile in women.

Vitamin D deficiency (<20 ng/mL, 78.5%) was prevalent in the patients in our study, even in summer (79.6%) and autumn (72.2%), in which sun exposure is high [12]. The prevalence of vitamin D deficiency was much higher than that in a prior study of T2DM patients in Xiamen, China (52.6%) [3]. This difference may partly reflect differences in sun exposure, which stimulates vitamin D production in the skin (Xiamen 24°N latitude vs. Xi'an 34°N latitude). These results suggest that T2DM patients, especially those in higher latitudes, should exercise outside, consume fatty fish, and take adequate amounts of vitamin D even in summer and autumn [13–15].

Previous studies linked high serum 25(OH)D levels with a low risk of metabolic disease [5]. Scragg et al. [7] found that 25(OH)D levels inversely correlated with insulin resistance in non-Hispanic whites and Mexican Americans but not African-Americans, which suggests that the correlation is ethnicity-dependent. Studies assessing this relationship in the Chinese population are limited [3, 9, 16–19]. Two studies in China identified low serum 25(OH)D concentration as a risk factor for insulin resistance and metabolic syndrome in T2DM patients in analyses adjusted for confounding factors including BMI [3, 17]. Conversely, another study in China found that 25(OH)D levels directly correlated with insulin resistance in men, but not women or both sex combined [10].

Lipoprotein(a) is an LDL-like protein and a potential risk factor for cardiovascular disease, especially in adults with impaired glucose metabolism [20, 21]. In the present study, serum 25(OH)D levels negatively correlated with lipoprotein(a) levels in men, and the association remained significant after adjusting for several confounding factors. GA-L is a better indicator of short-term glycemic control (about 2–3 weeks) than is HbA1c [22]; however, the percent reductions in HbA1c and GA-L levels were similar after insulin treatment in the study by Takahashi et al. [23]. We found that women in the high 25(OH)D group had lower GA-L levels than did those in the low 25(OH)D group. Serum 25(OH)D levels also correlated inversely with RBG levels in women, even after adjusting for BMI. Our results suggest that 25(OH)D preserves lipid and glucose metabolism in men and women with T2DM through different pathways, which may be linked to sex hormones.

The sex-specific association of 25(OH)D with metabolic risk factors may reflect differential effects of endogenous sex hormones and sex hormone-binding globulin (SHBG) on the risk factors [24]. Ding et al. [24] found that women with T2DM had higher testosterone levels, whereas men had lower levels of testosterone; SHBG levels inversely correlated with risk more strongly in women than in men. Furthermore, hyperandrogenic condition, such as polycystic ovarian syndrome in women, and hypoandrogenism in men have been strongly associated with cardiometabolic risk [24, 25]. Recent studies showed a significant sex-related relationship between the levels of 25(OH)D and several sex hormones, including SHBG, estradiol, and testosterone in adults [26, 27]. However, levels of neither sex hormones nor SHBG were determined in our study. Other sex-specific factors such as physical activity, smoking, fat distribution, and eating habits may also partly explain the sex-related contribution of 25(OH)D to T2DM development. Women subjects with overweight/obesity and higher frequency of smoking in men were observed to be significantly associated with higher incidence of metabolic syndrome and lower concentrations of 25(OH)D as well [15, 28, 29]. The associations of sex and 25(OH)D with metabolic risk factor were needed to be clarified in future studies.

The present study has several limitations. First, the amount of time the patients spent in the sun was not recorded. We did, however, use season as a proxy for sun exposure. Second, because this was a cross-sectional study, it is difficult to make causal inferences. Third, lifestyle factors such as physical activity and diet were not considered; this should be done in future studies. Finally, the sample size of this study was limited. Prospective studies with larger sample sizes are required to fully investigate the role of vitamin D in the development of T2DM. Despite the limitation, our study was the first to examine the relationships between serum 25(OH)D levels and metabolic risk factors on a sex-specific basis. It suggested that the protective effect of maintaining higher 25(OH)D levels against the development of T2DM may be through different mechanism in Chinese women and men.

## 5. Conclusions

Serum 25(OH)D levels negatively correlated with the levels of several metabolic risk factors in a sex-dependent manner in T2DM patients. Higher serum 25(OH)D levels indicated a more favorable lipid profile in men and a more favorable glucose profile in women. Sex-targeted intervention strategies of T2DM would be recommended.

## Figures and Tables

**Table 1:** Subject characteristics in 507 adults with type 2 diabetes according to sex.

Variable	Overall	Male	Female	*P* value
	*N* = 507	*N* = 321	*N* = 186	
Age (years)	59 (52–68)	57 (50–66)	63 (56–70)	**<0.001**
Weight (kg)	68 (60–77)	72 (65–80)	60 (55–68)	**<0.001**
Height (cm)	168 (162–174)	172 (168–176)	160 (156–165)	**<0.001**
BMI (kg/m^2^)	24.2 (22.3–26.2)	24.6 (22.5–26.2)	23.5 (21.8–26.1)	**0.026**
SBP (mmHg)	130 (120–144)	130 (120–146)	130 (120–142)	0.579
DBP (mmHg)	79 (70–86)	80 (72–87)	75 (70–83)	**<0.001**
ApoA (g/L)	1.1 (1.0–1.3)	1.1 (1.0–1.2)	1.2 (1.1–1.4)	**<0.001**
ApoB (g/L)	0.8 (0.6–0.9)	0.8 (0.6–0.9)	0.8 (0.6–0.9)	0.714
ApoE (mg/L)	32 (25–41)	30 (24–39)	34 (28–43)	**0.004**
Total-C (mmol/L)	4.0 (3.3–4.6)	3.8 (3.2–4.5)	4.1 (3.5–4.8)	**0.005**
HDL-C (mmol/L)	0.9 (0.8–1.2)	0.9 (0.8–1.1)	1.0 (0.9–1.2)	**<0.001**
LDL-C (mmol/L)	2.3 (1.8–2.8)	2.3 (1.7–2.8)	2.4 (1.9–3.0)	0.071
Lipoprotein(a) (mg/L)	107 (55–207)	107 (52–209)	109 (60–203)	0.800
Triglycerides (mmol/L)	1.4 (1.0–2.1)	1.4 (0.9–2.2)	1.3 (1.0–2.1)	0.645
ApoA/ApoB	1.4 (1.2–1.8)	1.4 (1.2–1.7)	1.5 (1.3–2.0)	**0.009**
HDL-C/LDL-C	0.4 (0.3–0.5)	0.4 (0.3–0.5)	0.4 (0.3–0.6)	0.204
PTH (pg/ml)	41 (32–54)	42 (32–55)	41 (31–54)	0.275
25(OH)D (ng/ml)	13.2 (9.2–19.0)	13.9 (9.7–19.3)	12.2 (8.5–17.9)	0.093
Calcium (mmol/L)	2.2 (2.1–2.3)	2.2 (2.1–2.3)	2.2 (2.1–2.3)	0.890
Phosphorus (mmol/L)	1.1 (1.0–1.2)	1.1 (1.0–1.2)	1.2 (1.0–1.3)	**0.001**
GA-L (%)^a^	20.9 (17.4–26.1)	20.9 (17.3–26.2)	20.9 (17.4–26.1)	0.959
HbA1c (%)	8.1 (6.7–9.7)	8.2 (6.8–9.9)	8.0 (6.6–9.6)	0.244
RBG (mmol/L)	7.1 (5.7–10.1)	7.1 (5.8–10.2)	6.8 (5.4–9.9)	0.406

Data are presented as median (IQR) values. BMI, body mass index; SBP, systolic blood pressure; DBP, diastolic blood pressure; ApoA, apolipoprotein A; ApoB, apolipoprotein B; ApoE, apolipoprotein E; Total-C, total cholesterol; HDL-C, high-density lipoprotein cholesterol; LDL-C, low-density lipoprotein cholesterol; PTH, parathyroid hormone; 25(OH)D, 25-hydroxyvitamin D; GA-L, glycated albumin; HbA1c, glycosylated hemoglobin; RBG, random blood glucose. ^a^*n* = 449.

**Table 2:** Association among sex 25(OH)D groups, and blood glucose and lipid profiles (*n* = 507).

	Male		Female		Sex	25(OH)D	Interaction
	Low 25(OH)D	High 25(OH)D	Low 25(OH)D	High 25(OH)D	*P*	*P*	*P*
N	145	174	104	81			
ApoA (g/L)	1.05 ± 0.22	1.11 ± 0.19	1.22 ± 0.26	1.22 ± 0.21	**<0.001**	0.098	0.105
ApoB (g/L)	0.79 ± 0.23	0.77 ± 0.20	0.80 ± 0.24	0.78 ± 0.23	0.070	0.534	0.964
ApoE (mg/L)	34.7 ± 16.5	33.8 ± 19.3	39.9 ± 18.0	34.3 ± 13.6	**0.001**	**0.033**	0.171
Lipoprotein(a) (mg/L)	195.3 ± 204.8	141.3 ± 137.9 ^*∗*^^†^	164.2 ± 202.9	202.4 ± 222.4	0.559	0.880	**0.010**
Total-C (mmol/L)	4.00 ± 1.15	3.97 ± 1.12	4.30 ± 1.24	4.17 ± 0.96	**<0.001**	0.665	0.690
HDL-C (mmol/L)	0.92 ± 0.26	0.98 ± 0.54	1.08 ± 0.40	1.10 ± 0.28	**<0.001**	0.083	0.441
LDL-C (mmol/L)	2.43 ± 1.07	2.28 ± 0.74	2.51 ± 1.00	2.46 ± 0.85	**0.004**	0.544	0.688
Triglycerides (mmol/L)	1.8 ± 1.4	2.8 ± 7.9	1.9 ± 1.6	2.4 ± 7.0	0.359	0.152	0.742
ApoA/ApoB	1.45 ± 0.61	1.53 ± 0.46	1.74 ± 1.41	1.71 ± 0.69	**0.032**	0.592	0.339
HDL-C/LDL-C	0.43 ± 0.23	0.47 ± 0.30	0.48 ± 0.24	0.50 ± 0.22	0.625	0.241	0.477
GA-L (%)^a^	22.5 ± 8.6	23.4 ± 8.7	23.3 ± 7.3	22.2 ± 8.8^*∗*†^	0.102	0.092	**0.023**
HbA1c (%)	8.5 ± 2.2	8.5 ± 2.2	8.5 ± 2.0	8.00 ± 2.1	0.136	**0.009**	0.091
RBG (mmol/L)	8.2 ± 4.5	8.9 ± 4.8	8.8 ± 5.1	7.6 ± 3.5	0.680	0.515	**0.028**

Data are presented as mean ± SD. ApoA, apolipoprotein A; ApoB, apolipoprotein B; ApoE, apolipoprotein E; Total-C, total cholesterol; HDL-C, high-density lipoprotein cholesterol; LDL-C, low-density lipoprotein cholesterol; 25(OH)D, 25-hydroxyvitamin D; GA-L, glycated albumin; HbA1c, glycosylated hemoglobin; RBG, random blood glucose. Data were analyzed using two-way analysis of covariance adjusted for age, BMI, season, marital status, smoking status, alcohol use behavior, insulin use, family history of diabetes, and diabetes duration. The levels of Apo B, Apo E, Total-C, HDL-C, LDL-C, lipoprotein(a), triglycerides, Apo A/Apo B, HDL-C/LDL-C, 25(OH)D, GA-L, HbA1c, and RBG were log-transformed for the analysis. ^a^*n* = 449. ^*∗*^*P* < 0.05 vs. female within the same 25(OH)D subjects. ^†^*P* < 0.05 vs. low 25(OH)D within the same sex group. Boldface indicates significance (*P* < 0.05).

**Table 3:** Multiple linear regression analysis with lipoprotein(a), GA-L, and RBC as dependent variables.

Variables	25(OH)D (ng/ml)					
	Model 1		Model 2		Model 3	
	*β*	*P*	*β*	*P*	*β*	*P*
Male						
Lipoprotein(a) (mg/L)	**−0.203**	**<0.001**	**−0.188**	**0.002**	**−0.185**	**0.002**
GA-L (%)	0.101	0.088	0.029	0.638	0.054	0.372
RBG (mmol/L)	0.07	0.211	0.058	0.331	0.054	0.366

Female						
Lipoprotein(a) (mg/L)	0.070	0.345	0.072	0.378	0.073	0.381
GA-L (%)	**−**0.077	0.331	**−**0.083	0.328	**−**0.135	0.118
RBG (mmol/L)	**−0.150**	**0.041**	**−0.189**	**0.016**	**−0.176**	**0.028**

25(OH)D, 25-hydroxyvitamin D; GA-L, glycated albumin; RBG, random blood glucose. The levels of lipoprotein(a), 25(OH)D, GA-L, and RBG were log-transformed for the analysis. *β*, standardized coefficient; linear regression model was used. Model 1 was unadjusted; Model 2 was adjusted for age, PTH, season, marital status, smoking status, alcohol use behavior, insulin use, family history of diabetes, and diabetes duration; Model 3 included terms for Model 2 and BMI. ^a^*n* = 449. Boldface indicates significance (*P* < 0.05).

## Data Availability

The data used to support the findings of this study are available from the corresponding author upon request.

## Supplementary Materials

Supplementary Materials Comparison of the vitamin D status according to sex groups.
