# Supplementary material for: Sex-Specific Association between Serum 25-Hydroxyvitamin D and Metabolic Risk Factors in T2DM Patients
Source: Int J Endocrinol. 2020 Jun 4;2020:9238719. doi: 10.1155/2020/9238719 (PMC7292989; doi:10.1155/2020/9238719)
Supplement: Supplementary Materials — Comparison of the vitamin D status according to sex groups. [file 9238719.f1.pdf]

Supplemental table 1: Comparison of the vitamin D status according sex groups.

	Deficiency N (%)	Insufficiency N (%)	Sufficiency N (%)
Male	83 (25.9)	167 (52.0)	71 (22.1)
Female	64 (34.4)	84 (45.2)	38 (20.4)
Total	147 (29.0)	251 (49.5)	109(21.5)

Vitamin D status defined as: Deficiency < 10ng/mL; insufficiency ≧10 ng/mL to < 20 ng/mL; and sufficiency ≧20 ng/ml.

$\chi 2$=4.247, P_{trend} =0.120
